# Fibrotic Changes Depicted by Thin-Section CT in Patients With COVID-19 at the Early Recovery Stage: Preliminary Experience

**DOI:** 10.3389/fmed.2020.605088

**Published:** 2020-11-24

**Authors:** Zhen Lu Yang, Chong Chen, Lu Huang, Shu Chang Zhou, Yu Na Hu, Li Ming Xia, Yan Li

**Affiliations:** Department of Radiology, Tongji Hospital, Tongji Medical College, Huazhong University of Science and Technology, Wuhan, China

**Keywords:** coronavirus disease 2019 (COVID- 19), fibrotic change, chest CT imaging, follow-up, risk factors

## Abstract

**Objectives:** To analyze follow-up CTs of patients recovering from COVID-19 in Wuhan, focusing on fibrotic change and its relevant risk factors.

**Methods:** From January 13 to February 27, 2020, 166 hospitalized patients meeting our criteria were included. The scores of fibrotic patterns on follow-up CT were evaluated. Patients were designated as group 1 (with CT evidence of fibrotic pattern) and group 2 (without CT evidence of fibrotic pattern). Multivariate logistic regression was performed to explore risk factors for fibrotic change in patients with COVID-19.

**Results:** The follow-up CTs were obtained on 56 days (median, IQR 51–63 days) after symptom onset. Of the 166 patients (mean age, 57 ± 15 years; 69/166 male), 46% (76/166) had CT evidence of fibrotic change and 77% (127/166) were severe or critical cases. Among patients with fibrotic change on CT, 84% (64/76) got a minimal or mild score of fibrosis. The high total score on peak CT, peak eosinophils, erythrocyte sedimentation rate (ESR) and advancing age were related to lung fibrotic change in patients with COVID-19.

**Conclusion:** Forty six percentages of patients (mainly severe or critical cases) with COVID-19 showed fibrotic change on follow-up CT at early recovery phase, while the extent of fibrosis was not large. The advancing age, high total score on peak CT, peak eosinophils and ESR were associated with fibrotic change depicted by CT in patients recovering from COVID-19. An extended follow up by CT imaging and pulmonary function testing is necessary to fully assess the sequela of COVID-19.

## Highlights

- In our cohort, 77% (127/166) patients were severe or critical cases and 46% (76/166) patients with COVID-19 presented with fibrotic patterns on follow-up CT at early recovery phase (56 days after symptom onsets).
- The scores of fibrotic changes in 84% (64/76) patients were estimated as minimal or mild.
- The high total score on peak CT, peak eosinophils, erythrocyte sedimentation rate (ESR) and advancing age were related to lung fibrotic change depicted by CT in patients with COVID-19.

## Introduction

Coronavirus disease 2019 (COVID-19) caused by severe acute respiratory syndrome coronavirus 2 (SARS-CoV-2), started in December 2019 and rapidly reached a global pandemic (1, 2). COVID-19 is highly infectious and has a high mortality rate in elderly patients or those with comorbidities (3, 4). By September 11, 2020, there are more than 27 million confirmed cases and 899,916 deaths globally according to reports of World Health Organization (WHO) (5).

Since the outbreak of COVID-19, many studies have been conducted to reveal the epidemiological features, symptoms/signs, laboratory data, radiographic findings and treatments of this disease to facilitate the diagnosis and management of patients (6–11). Chest CT is critical in early diagnosis, dynamic observation, treatment evaluation and prognostic prediction (12, 13), and its patterns have been well-described in the acute and early recovery phases (2, 7, 8, 14, 15).

Previous literatures reported that 62% of patients with Severe Acute Respiratory Syndrome (SARS) and 33% patients with Middle East Respiratory Syndrome (MERS) showed evidences of lung fibrosis after recovery (16, 17). Those suggested that fibrotic changes may develop as a crucial complication in patients with COVID-19 (18). However, follow-up information and evidence of lung fibrosis in patients recovering from COVID-19 is rare at present. Wuhan city of China is the early pandemic center of COVID-19 and has accumulated huge recovery cases as well as corresponding imaging data. Therefore, the purpose of this study is to report the follow-up CT findings of patients recovering from COVID-19 in Wuhan, with focus on the lung fibrotic patterns and its relevant risk factors; this may make sense for understanding disease course and better managing infected patients.

## Materials and Methods

### Patients and Data Collection

**Figure 1:**
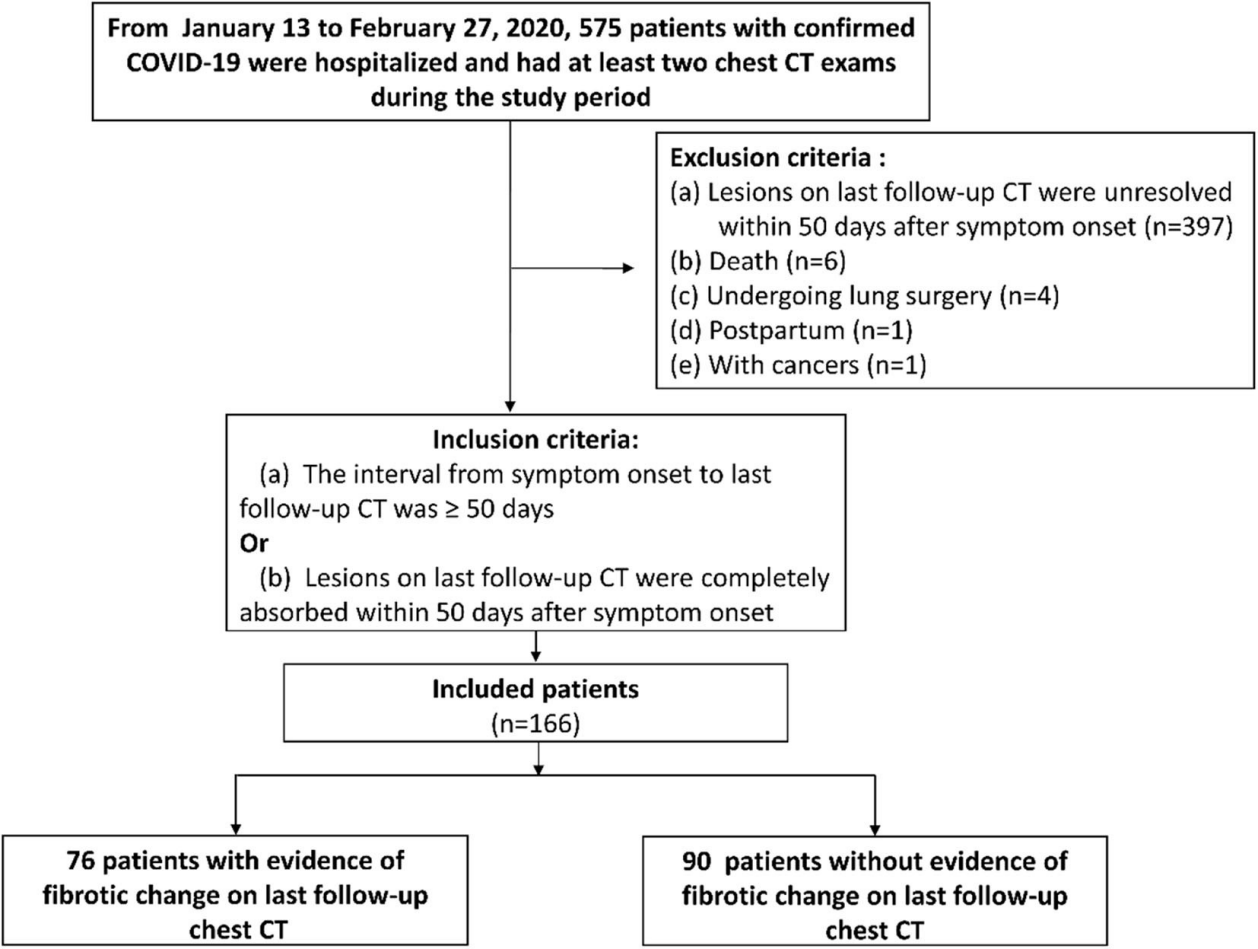
The flowchart of this study. COVID-19, coronavirus disease 2019.

This retrospective study was approved by our institutional review board (IRB number of TJ-C20200141) with a waived informed consent of patients. From January 13 to February 27, 2020, a total of 575 patients (mean age, 60 ± 14 years; 46% [267/575] male) with confirmed COVID-19 according to diagnostic criteria of WHO (19) were hospitalized in our hospital and had at least two chest CT exams during the study period. Patients were included when meeting one of the following inclusion criteria (Figure 1): (a) The interval from symptom onset to last follow-up CT was more than 50 days no matter whether lesions were absorbed or not [we set 50 days as the cut-off value for inclusion criterion based on the time when the fibrosis appeared on chest CT of SARS and MERS patients (16, 17)]; or (b) Lung lesions on last follow-up CT were completely absorbed within 50 days after symptom onset. The exclusion criteria were (Figure 1): (a) Lesions were unresolved on last follow-up CT within 50 days after symptom onset (*n* = 397); (b) Postpartum (*n* = 1); (c) With cancers (*n* = 1); (d) Death during hospitalization (*n* = 6); (e) Undergoing lung surgery (*n* = 4) because of potential inflammation or hemorrhage. As result, 166 patients were included in this study.

All of the 166 patients were discharged at the beginning of this study. The discharge criteria were provided by National Health Commission of China (20): (a) No fevers for more than 3 days; (b) Significantly alleviated respiratory symptoms; (c) Radiographic abnormalities on chest radiograph or CT were improved; (d) two consecutive nucleic acid assays with respiratory samples were negative for SARS-CoV-2 with an interval of at least 24 h.

We recorded all available clinical, laboratory and treatment information of patients extracted from clinical electronic medical records and nursing records in our hospital information system (HIS).

### Chest CT Imaging Protocols and Interpretation

The CT systems and imaging parameters used in this work were described in our previous study (21). All images were obtained on one of the three CT systems (Siemens Healthineers, Germany Optima 660, GE, America; uCT 780, United Imaging, China; Somatom Definition AS+,) with patients in supine position. The main scanning parameters were as follow: tube voltage = 120 kVp, automatic tube current modulation (30–70 mAs), slice thickness = 0.625–1.250 mm, field of view = 350 mm.

For each one patient, a total of two scans needed to be evaluated (the peak chest CT and last follow-up chest CT). Two radiologists (with 8 and 3 years of experience in interpreting chest CT imaging, respectively) reviewed all images by consensus. The chest CT scan with the most severe changes, which was defined as peak chest CT, was determined by comparing series of CT scans of one patient.

For peak chest CT, we assessed radiologic abnormities, including ground-glass opacity (GGO), consolidation, reticulation/thickened interlobular septa, lung distortion and nodular. The severity of each lung lobe (left upper/lower lobe, right upper/middle/lower lobe) was scored on basis of lesion extent in the corresponding lobe, as 0 (0%), 1 (1–5%), 2 (6–24%), 3 (25–49%), 4 (50–74%), and 5 (75–100%) (13) (Table 1). Total score of five lobes of each patient was summed with 0 as none, 1–5 as minimal, 6–10 as mild, 11–15 as moderate, 16–20 as severe, 21–25 as critical.

**Table 1:** CT scoring system of lesion extent on peak CT and fibrosis extent on last follow-up CT.

**Lesion extent or fibrosis extent of a lobe**	**CT score of a lobe**
0	0
1–5%	1
6–24%	2
25–49%	3
50–74%	4
75–100%	5

As to last follow-up chest CT images, we evaluated the evidence of fibrotic changes, including parenchymal bands, traction bronchiectasis, irregular interfaces, lung distortion, and honeycombing (16, 22–24). Lung distortion was defined as anatomical displacement of bronchus, vessel and interlobular septum of lung. Traction bronchiectasis was defined as dilation of bronchus and irregularity of bronchial wall. The lesion extent of each lung lobe was scored as 0 (0%), 1 (1–5%), 2 (6–24%), 3 (25–49%), 4 (50–74%), and 5 (75–100%) (23, 24) (Table 1). Total fibrosis score of five lobes of each patient was summed with 0 as none, 1–5 as minimal, 6–10 as mild, 11–15 as moderate, 16–20 as severe, 21–25 as critical. The distribution of fibrotic patterns was recorded as: predominantly peripheral, peribronchovascular, or random. The residual GGO and consolidation on last follow-up CT were also recorded.

According to evaluation of fibrotic changes on CT, patients were classified as group 1 (with CT evidence of fibrotic pattern) and group 2 (without CT evidence of fibrotic pattern) for analysis.

### Statistical Analysis

The statistical analysis of this study was performed using Python (version 3.8.1). When *P*-value was ≤ 0.05, a significantly statistical difference was considered. Continuous variables were displayed as median with interquartile range (IQR) or means ± standard deviations, and compared using Mann-Whitney U test; Categorical data was shown as counts and percentages in each category, and compared by Chi-square test between group 1 and group 2. The boxplots were used to show distribution of laboratory testing results. Multivariate logistic regression was performed by forward stepwise (likelihood ratio) to explore risk factors for fibrotic changes in patients with COVID-19.

## Results

### General Characteristics of Patients

The last follow-up CT images were obtained on 56 days (median, IQR 51–63 days) after symptom onset and the median interval from onset to peak CT was longer in group 1 than in group 2 (23 vs. 18 days, *p* = 0.001) (Table 2).

**Table 2:** Characteristics and treatments of patients with COVID-19.

	**Total patients (*n* = 166)**	**With CT evidence of fibrosis (group 1, *n* = 76)**	**Without CT evidence of fibrosis (group 2, *n* = 90)**	***P*-value**
**Age (years)**				
Mean (Range)	57 ± 15 (14–89)	63 ± 11 (28–86)	52 ± 16 (14–89)	<0.001
**Male sex**	69 (42%)	37 (49%)	32 (36%)	0.087
**Time interval (median, days)**				
From onset of symptom to peak CT	20 (12–26)	23 (16–28)	18 (11–24)	0.001
From onset of symptom to follow-up CT	56 (51–63)	58 (54–66)	54 (35–59)	<0.001
**Severity of illness**				
Moderate	39 (23%)	10 (13%)	29 (32%)	<0.001
Severe	113 (68%)	54 (71%)	59 (66%)	
Critical	14 (8%)	12 (16%)	2 (2%)	
**Treatments**				
Oxygen support	145/166 (87%)	72/76 (95%)	73/90 (81%)	0.009
Nasal cannula or face mask	137/166 (83%)	64/76 (84%)	73/90 (81%)	0.600
Noninvasive mechanical ventilation	7/166 (4%)	7/76 (9%)	0/90 (0%)	0.011
Invasive mechanical ventilation	1/166 (1%)	1/76 (1%)	0/90 (0%)	0.458
Glucocorticoids (methylprednisolone)	75/166 (45%)	50/76 (66%)	25/90 (28%)	<0.001
Immunoglobulin	57/166 (34%)	35/76 (46%)	22/90 (24%)	0.003
Antiviral agents	159/166 (96%)	75/76 (99%)	84/90 (93%)	0.186
Antibiotics	110/166 (66%)	63/76 (83%)	47/90 (52%)	<0.001
Antifungal agents	2/166 (1%)	2/76 (3%)	0/90 (0%)	0.208
ECMO	0/166 (0%)	0/76 (0%)	0/90 (0%)	1.000
CRRT	0/166 (0%)	0/76 (0%)	0/90 (0%)	1.000

*Data are n (%) or median (IQR). Mean age is mean ± standard deviation. COVID-19, coronavirus disease 2019; ECMO, extracorporeal membrane oxygenation; CRRT, continuous renal replacement therapy*.

Of 166 included patients (mean age, 57 ± 15 years, 42% [69/166] male), 76 patients (mean age, 63 ± 11 years, 49% [37/76] male) were designated as group 1 (with CT evidence of fibrotic pattern) and 90 patients (mean age, 52 ± 16 years, 36% [32/90] male) were designated as group 2 (without CT evidence of fibrotic pattern) (Table 2, Figure 1). According to Diagnosis and Treatment Protocol for Novel Coronavirus Pneumonia (Trial Version 7) (20), there were 127/166 (77%) severe or critical cases in our cohort (Table 2). Patients in group 1 were older and severer than in group 2 (both *p* < 0.001). More patients in group 1 received non-invasive mechanical ventilation, glucocorticoids (methylprednisolone) and antibiotics than those in group 2 (all *p* < 0.05) (Table 2).

### Laboratory Examinations

**Figure 2:**
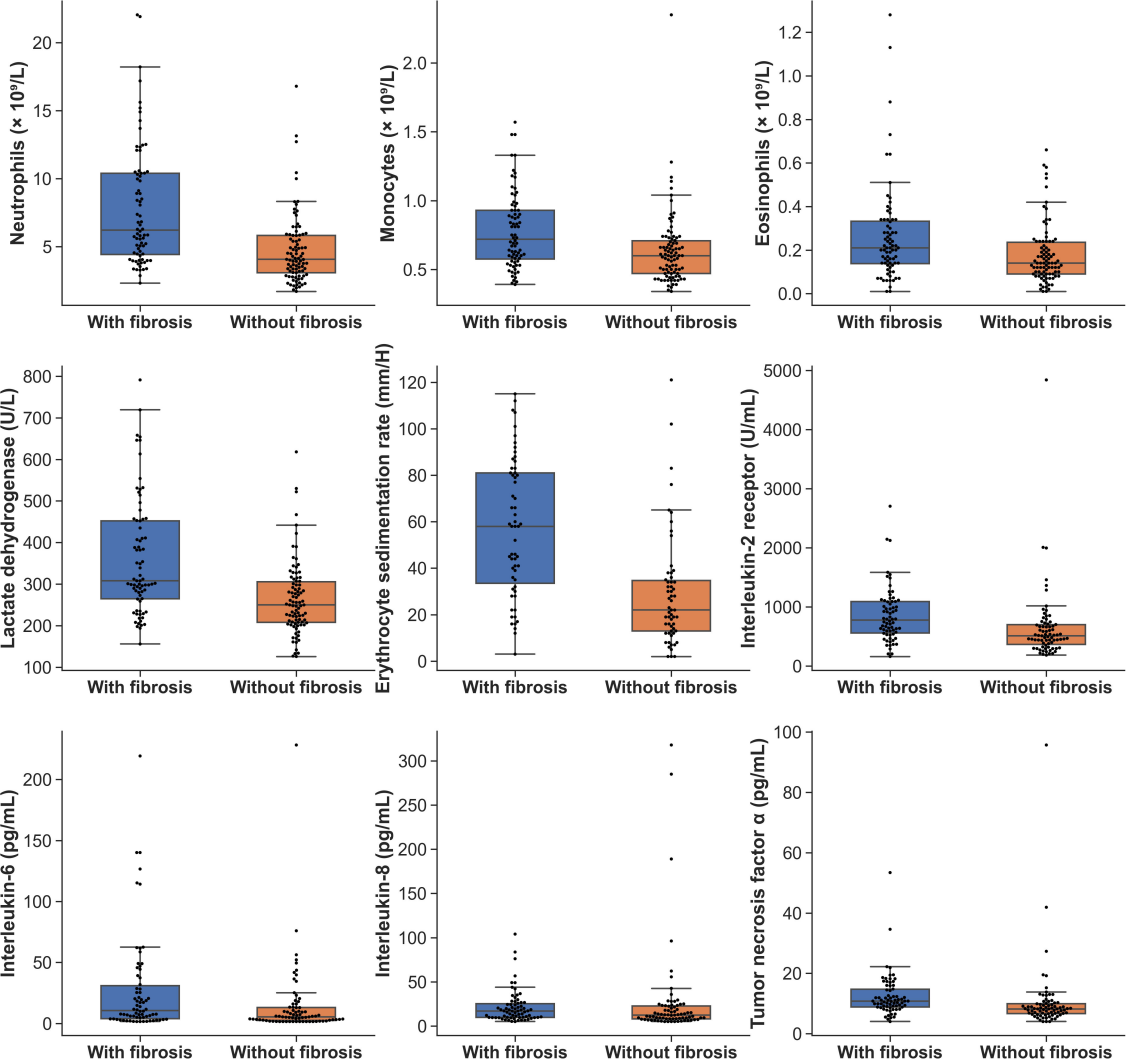
The boxplots of laboratory parameters of patients in the two groups.

The peak laboratory data of patients during hospitalization was analyzed. Higher levels of leucocytes, neutrophils, eosinophils, monocytes counts, lactate dehydrogenase, erythrocyte sedimentation rate (ESR) and C-reactive protein were found in patients of group 1 when compared with group 2 (Table 3) (all *p* < 0.01). As to cytokines testing, the interleukin-2 receptor (IL-2R), interleukin-6 (IL-6), interleukin-8 (IL-8) and tumor necrosis factor α (TNF-α) of patients in group 1 were significantly greater than that of patients in group 2 (all *p* < 0.05) (Table 3). The boxplots of laboratory parameters were displayed in Figure 2.

**Table 3:** Peak laboratory data of patients with COVID-19.

	**Normal range**	**Total patients**	**With CT evidence of fibrosis**	**Without CT evidence of fibrosis**	***P*-value**
		**(*n* = 166)**	**(group 1, *n* = 76)**	**(group 2, *n* = 90)**	
**White blood cells**					
Leucocytes (×10^9^/L)	3.5–9.5	7.25 (5.63–9.75)	8.52 (6.45–12.49)	6.55 (5.43–8.01)	<0.001
Increased		45/166 (27%)	33/76 (43%)	12/90 (13%)	<0.001
Neutrophils (×10^9^/L)	1.8–6.3	4.92 (3.55–7.46)	6.20 (4.42–10.39)	4.05 (3.08–5.82)	<0.001
Increased		52/166 (31%)	36/76 (47%)	16/90 (18%)	<0.001
Lymphocytes (×10^9^/L)	1.1–3.2	1.82 (1.36–2.25)	1.73 (1.23–2.16)	1.85 (1.44–2.28)	0.125
Increased		4/166 (2%)	3/76 (4%)	1/90 (1%)	0.497
Eosinophils (×10^9^/L)	0.02–0.52	0.17 (0.10–0.26)	0.21 (0.14–0.33)	0.14 (0.09–0.23)	0.003
Increased		11/166 (7%)	6/76 (8%)	5/90 (6%)	0.546
Monocytes (×10^9^/L)	0.1–0.6	0.64 (0.50–0.83)	0.72 (0.57–0.93)	0.60 (0.47–0.71)	<0.001
Increased		93/166 (56%)	51/76 (67%)	42/90 (47%)	0.008
**Cytokines**					
Interleukin-1 β (pg/mL)	<5	5.00 (5.00–5.30)	5.00 (5.00–5.30)	5.00 (5.00–5.15)	0.771
Increased		40/145 (28%)	19/70 (27%)	21/75 (28%)	0.908
Interleukin-2 receptor (U/mL)	223–710	616.00 (440.00–917.00)	777.00 (557.50–1090.50)	511.00 (365.50–698.00)	<0.001
Increased		56/145 (39%)	39/70 (56%)	17/75 (23%)	<0.001
Interleukin-6 (pg/mL)	<7.0	7.21 (3.11–20.38)	10.61 (3.93–31.02)	5.36 (2.45–13.14)	0.003
Increased		76/145 (52%)	45/70 (64%)	31/75 (41%)	0.006
Interleukin-8 (pg/mL)	<62	13.60 (9.20–23.70)	16.95 (9.70–25.38)	12.30 (8.10–22.70)	0.039
Increased		8/145 (6%)	3/70 (4%)	5/75 (7%)	0.792
Interleukin-10 (pg/mL)	<9.1	5.00 (5.00–7.70)	5.00 (5.00–9.15)	5.00 (5.00–5.90)	0.103
Increased		27/145 (19%)	18/70 (26%)	9/75 (12%)	0.034
Tumor necrosis factor α (pg/mL)	<8.1	9.20 (7.50–11.90)	10.80 (8.85–14.73)	8.20 (6.65–9.95)	<0.001
Increased		96/145 (66%)	56/70 (80%)	40/75 (53%)	0.001
**Other biomarkers**					
Lactate dehydrogenase (U/L)	135–225	283.00 (224.25–350.50)	308.00 (264.75–452.25)	250.00 (208.25–305.50)	<0.001
Increased		126/166 (76%)	68/76 (89%)	58/90 (64%)	<0.001
Erythrocyte sedimentation rate (mm/H)	0.00–15.00	35.00 (19.00–64.00)	58.00 (33.50–81.00)	22.00 (13.00–34.75)	<0.001
Increased		89/117 (76%)	55/59 (93%)	34/58 (59%)	<0.001
C-reactive protein (mg/L)	<1.0	22.80 (4.60–72.50)	61.70 (15.80–135.55)	11.25 (2.47–33.62)	<0.001
Increased		151/166 (91%)	75/76 (99%)	76/90 (84%)	0.001

*Data are median (IQR) or n/N (%), where N is the total number of patients with available data. COVID-19, coronavirus disease 2019*.

### Image Findings on Peak and Last Follow-Up CTs

**Figure 3:**
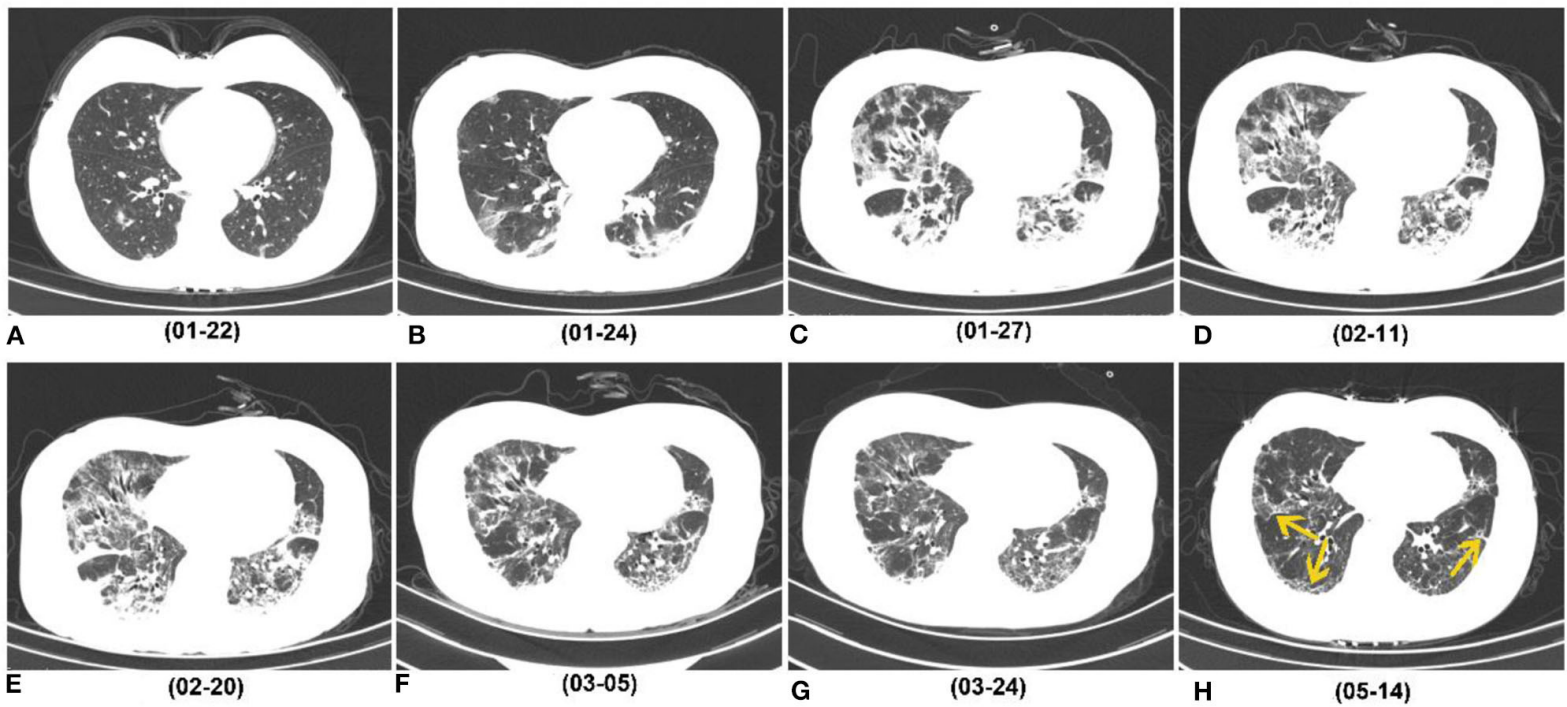
Series of follow-up CTs of a 40-year-old woman with COVID-19 who caught a fever on January 19, 2020 and was admitted at hospital on January 27, 2020. **(A)** Initial chest CT on January 22 (3 days after symptoms onset) showed sporadic GGO in bilateral lower lobes. **(B–E)** Two days later, consolidation appeared. Whereafter, the extent of GGO and consolidation enlarged, and reached its peak on February 11, and remained the peak level until February 20. **(F,G)** At the recovery stage, lesions were gradually absorbed. **(H)** On May 14 (116 days after symptoms onset), most consolidations were absorbed, while irregular interfaces and parenchymal bands were observed as evidence of fibrotic change (yellow arrow). COVID-19, coronavirus disease 2019; GGO, ground-glass opacity.

On last follow-up CTs, 76/166 (46%) patients showed CT evidences of fibrotic changes, manifesting as parenchymal bands (76% [58/76]), irregular interfaces (32% [24/76]) (Figure 3), traction bronchiectasis (38% [29/76]), lung distortion (25% [19/76]), and honeycombing (9% [7/76]). The fibrotic patterns principally showed peripheral distribution (89% [68/76]). The total scores of fibrotic changes presented as minimal or mild (1–10 points) in 84% (64/76) patients (Table 4). One patient was estimated as severe and no critical score were given. Besides, a total of 51 patients showed residual GGO or consolidation on the last follow-up CTs.

**Table 4:** Radiographic findings on peak chest CT and features of fibrotic change on last follow-up CT in patients with COVID-19.

	**Total patients**	**With CT evidence of fibrosis**	**Without CT evidence of fibrosis**	***P*-value**
	**(*n* = 166)**	**(group 1, *n* = 76)**	**(group 2, *n* = 90)**	
**Radiographic features on peak chest CT**				
Manifestations on peak chest CT				
GGO	154/166 (93%)	71/76 (93%)	83/90 (92%)	0.766
Consolidation	128/166 (77%)	69/76 (91%)	59/90 (66%)	<0.001
Reticulation/thickened interlobular septa	38/166 (23%)	31/76 (41%)	7/90 (8%)	<0.001
Lung distortion	26/166 (16%)	23/76 (30%)	3/90 (3%)	<0.001
Nodular lesions	6/166 (4%)	3/76 (4%)	3/90 (3%)	0.837
Distribution of lesions on peak CT
1–4 lung lobes	58/166 (35%)	9/76 (12%)	49/90 (54%)	<0.001
5 lung lobes	108/166 (65%)	67/76 (88%)	41/90 (46%)	
Total scores on peak chest CT	12 (6–17)	16 (12–20)	8 (4–12)	<0.001
0 (None)	2/166 (1%)	0/76 (0%)	2/90 (2%)^*^	
1–5 (Minimal)	26/166 (16%)	2/76 (3%)	24/90 (27%)	<0.001
6–10 (Mild)	47/166 (28%)	11/76 (14%)	36/90 (40%)	
11–15 (Moderate)	34/166 (20%)	18/76 (24%)	16/90 (18%)	
16–20 (Severe)	37/166 (22%)	28/76 (37%)	9/90 (10%)	
21–25 (Critical)	20/166 (12%)	17/76 (22%)	3/90 (3%)	
**Features of fibrotic change on last follow-up CT**				
Manifestations of fibrotic change on last follow-up CT				
Parenchymal bands	58/166 (35%)	58/76 (76%)		
Irregular interfaces	24/166 (14%)	24/76 (32%)		
Traction bronchiectasis	29/166 (17%)	29/76 (38%)		
Lung distortion	19/166 (11%)	19/76 (25%)		
Honeycombing	7/166 (4%)	7/76 (9%)		
Distribution of fibrotic change on last follow-up CT				
Peripheral	68/166 (41%)	68/76 (89%)		
Peribronchovascular	5/166 (3%)	5/76 (7%)		
Random	3/166 (2%)	3/76 (4%)		
Total scores of fibrotic pattern on last follow-up CT				
1–5 (Minimal)	45/166 (27%)	45/76 (59%)		
6–10 (Mild)	19/166 (11%)	19/76 (25%)		
11–15 (Moderate)	11/166 (7%)	11/76 (14%)		
16–20 (Severe)	1/166 (1%)	1/76 (1%)		
21–25 (Critical)	0 (0%)	0 (0%)		

*Data are n (%) or median (IQR). COVID-19, coronavirus disease 2019; GGO, ground-glass opacity*.

* *The two patients presented with severe and persistent respiratory symptom, however, all CT images of them during hospitalization were negative. The CTs performed in the severest clinical conditions were defined as peak chest CTs of the two patients*.

**Figure 4:**
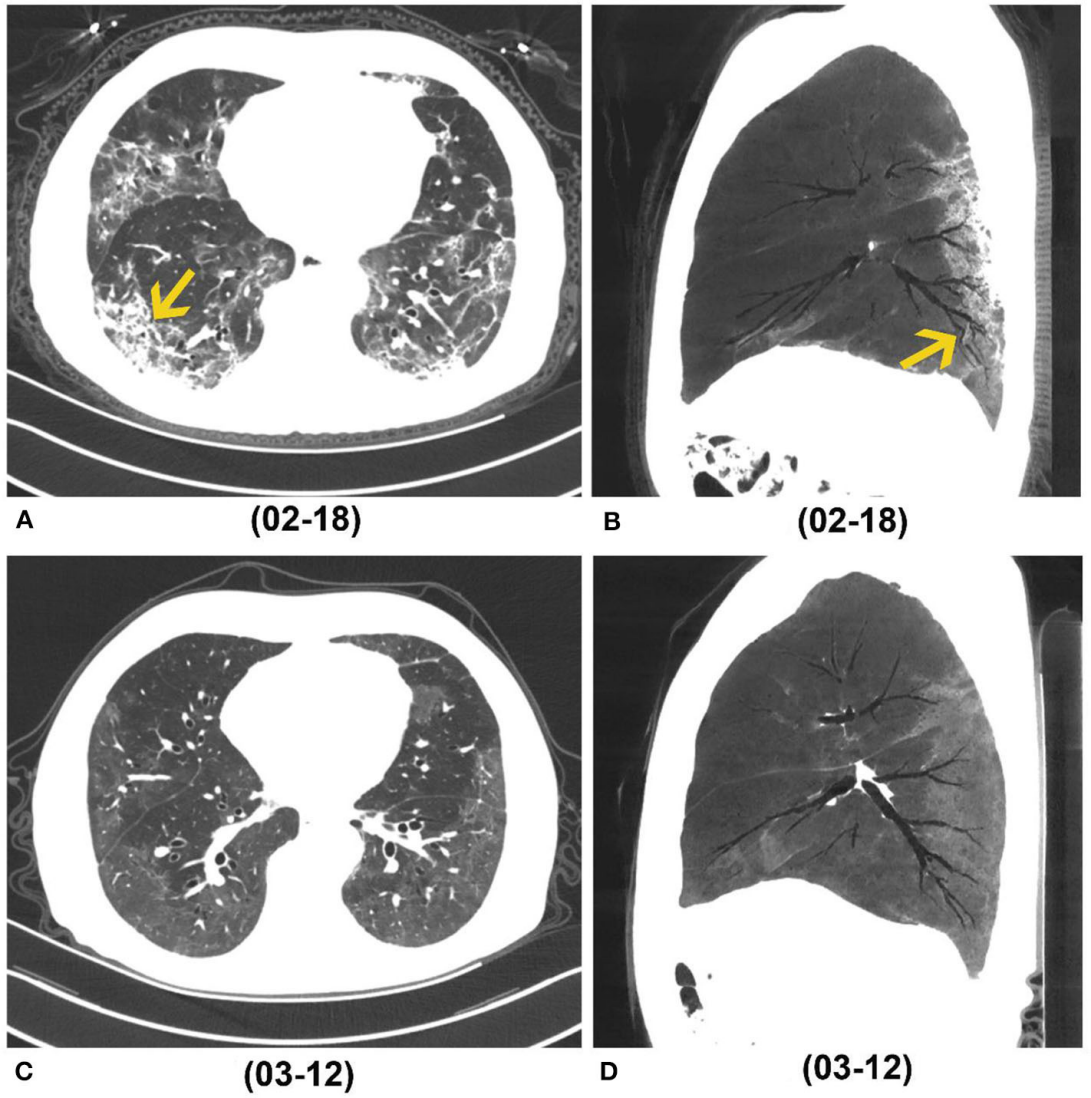
A 68-year-old man who caught a fever and cough on January 17, 2020. **(A,B)** Axial and sagittal CT (Min IP) performed on February 18 (peak CT, 32 days after symptoms onset) showed GGO, reticulation (yellow arrow in A) and bronchial distortion (yellow arrow in B) in the bilateral lungs. **(C,D)** Axial and sagittal CT (Min IP) performed on March 12 (last follow-up CT, 55 days after symptoms onset) showed residual GGO, while previous reticulation and distortion disappeared. Min IP, minimum intensity projection; COVID-19, coronavirus disease 2019; GGO, ground-glass opacity.

On peak CT, the main imaging features were GGO (154/166), consolidation (128/166) and reticulation/thickened interlobular septa (38/166) (Table 4, Figure 3). More patients presented with consolidation, reticulation/thickened interlobular septa and lung distortion in group 1 than did patients in group 2 (all *p* < 0.001) (Table 4). A higher total score on peak CT was found in group 1 (median, 16) than group 2 (median, 8) (*P* < 0.001). Lung distortion (a sign of fibrotic change) was found in 26 patients on their peak CTs, persisted in 13 patients and recovered in 13 patients on their last follow-up CTs (Figure 4).

### Risk Factors of Fibrotic Change in Patients With COVID-19

According to multivariate logistic regression analysis, high total score on peak CT, eosinophils count, ESR and advancing age were related to fibrotic change on CT at early recovery stage in patients with COVID-19, with the odds ratios (ORs) of 1.156, 43.250, 1.024, and 1.048, respectively (all *p* < 0.05) (Table 5).

**Table 5:** Significant risk factors for fibrotic change depicted by CT in patients with COVID-19.

	**B**	**SE**	***P*-value**	**OR**	**95% CI for the OR**
Total score on peak chest CT	0.145	0.048	0.003	1.156	1.052–1.271
Eosinophils	3.767	1.633	0.021	43.250	1.760–1062.706
Erythrocyte sedimentation rate	0.024	0.010	0.015	1.024	1.005–1.045
Age	0.047	0.019	0.014	1.048	1.009–1.087

*B, regression coefficient; SE, standard error; OR, odds ratio; CI, confidence intervals; COVID-19, coronavirus disease 2019*.

## Discussion

The investigation of fibrosis that may correlate with pulmonary functional changes is beneficial for better management of patients with COVID-19. However, relevant evidence has not been reported. In our study, 46% (76/166) patients presented with fibrotic changes on follow-up CT during early recovery phase (56 days after symptom onsets), and the extent of fibrotic changes in 84% (64/76) patients was minimal or mild. According to multivariate logistic regression, high total score on peak CT, peak eosinophils, ESR and advancing age were related to fibrotic changes depicted by CT in patients recovering from COVID-19.

Fibrotic patterns on CT occurred in less than half (46%, 76/166) of patients in this study, which is lower than that of patients with SARS (62%) (16) and higher than that of patients with MERS (32%) (17). In our cohort, 77% were severe or critical cases, therefore, this data may more reflect the proportion of fibrotic change in critically ill patients. Though many patients showed CT evidence of fibrotic change at recovery phase, the extent of fibrotic pattern was not large. We speculated those fibrotic changes may not put a great impact on the clinical symptom and life quality, however, this should be validated through pulmonary function tests.

**Figure 5:**
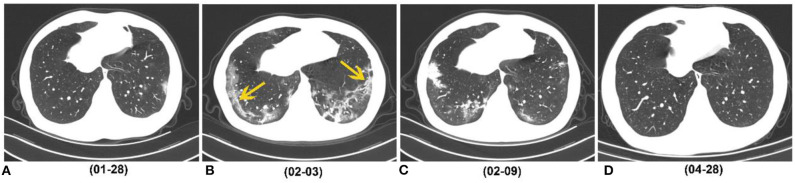
A 29-year-old man who caught a fever on January 23, 2020, received three follow-up scans after the initial chest CT. **(A)** Initial CT performed on January 23 (the day of symptoms onset) showed patchy GGO in the left lower lobe. **(B)** Chest CT performed on February 3 showed consolidation, GGO and strip-like lesions (easily misdiagnosed as fibrosis) in the bilateral lower lobes (yellow arrow). **(C)** Chest CT performed on February 9 showed strip-like lesions disappeared, and multiple GGO and consolidation presented in the bilateral lower lobes. **(D)** On April 28 (116 days after symptoms onset), CT image showed that all lesions were absorbed. COVID-19, coronavirus disease 2019; GGO, ground-glass opacity.

The median interval from onset to peak CT was longer in group 1 than in group 2, which indicated that patients with evidence of fibrotic change may have a longer and more serious lung infections, which is similar to the findings of previous study (25). In this study, fibrotic pattern depicted by CT in most patients (89%, 68/76) had peripheral distribution, which was consistent with the predominant distribution of GGO and consolidation and suggested the recovery process of inflammation. The lung distortion (a sign of fibrosis) showed in 16% (26/166) patients on peak CT with 50% (13/26) existing persistently to the last follow-up CT and 50% (13/26) resolving (Figure 4). The median interval between symptom onsets and peak CT was 20 days. These findings suggested that patients with COVID-19 may develop lung fibrotic signs at an early stage and early fibrosis may be, at least partly, reversible (26) (Figure 5). Besides fibrosis, bronchial dilation, as a radiographic sign, can also be seen in the setting of acute/subacute inflammation, coughing or barotrauma (27, 28), and can be reversible. This may partly explain some reversible cases in our study. By the end of this study, there were still many patients with GGO unresolved. Persistent GGO may represent residual inflammation that may resolve or evolve to fibrosis over time. Therefore, longer follow-up time was needed for observing fibrosis changes and its impact on respiratory symptoms.

Several risk factors of fibrotic change in patients with COVID-19 were identified. Larger lesions extent and higher total CT score on peak CT reflect severer lung injury and inflammation in patients. During persistent or recurrent injury and repair, fibrosis can develop more commonly (26). It is effective and convenient to evaluate disease and predict fibrotic change in patients with COVID-19 using dynamic CTs to better manage infected patients.

A higher level of eosinophils count in patients with evidence of fibrotic change was observed in our study. Previous studies reported eosinophils could produce essential pro-fibrotic cytokines or chemokines, such as transforming growth factor β1 (TGF-β1), platelet-derived growth factor (PDGF), IL-13 and chemokine (C-C motif) ligand 18 (CCL18) (25, 29, 30). Based on our results, eosinophils count was a risk factor for fibrotic change with a high ORs (43.250), which suggested eosinophils count might be used to predict the fibrotic change in patients with COVID-19. IL-2R, 6, 8, and TNF-α are important pro-inflammatory cytokines, which may promote non-specific immune response and cause pulmonary injury (9). The results of the relative higher IL-2R, 6, 8, and TNF-α levels in patients with CT evidence of fibrosis support the points that those cytokines play a vital role in the inflammatory response and driving fibrosis in injured tissues. However, much still need to be learned about the mechanism of fibrosis changes caused by this highly infectious disease.

Mechanical stress induces lung fibrosis, and epithelia-mesenchymal transition may play an important role in mediating the ventilator-induced lung fibrosis (31). In our cohort, the proportion of patients with non-invasive mechanical ventilation treatment in group 1 (with CT evidence of fibrotic change) (9%, 7/76) was higher than that of patients in group 2 (0/90) (*p* = 0.011). However, it is difficult to assess the impact of mechanical ventilation on fibrosis formation because patients who need ventilator treatment always have severe pulmonary inflammation.

There are some limitations in our study. First, selected bias may have been involved due to the retrospective nature. Second, there is no pathologic confirmation of fibrosis in patients of this study. Third, true traction bronchiectasis caused by fibrotic tissue is non-reversible. In our study, bronchial dilation on CT images was recorded as a radiographic sign of fibrosis, which may appear confusing because this sign can be seen in acute/subacute inflammation and may be reversible and does not necessarily correspond to underlying fibrosis. Therefore, longer follow-up time and comprehensive analysis by considering clinical symptoms, laboratory exams and dynamic changes on CTs are necessary when evaluating pulmonary fibrosis on CT images. Lastly, it is better to assess fibrosis changes after a longer follow-up period, because GGO did not completely resolve in some patients by the end of our study. Our results summarized the preliminary and overall follow-up CT findings in patients at the early recovery stage of this disease.

In conclusion, in our study, 46% patients (mainly severe or critical cases) with COVID-19 have CT evidences of fibrotic pattern during early recovery phase, while the extent of fibrotic change was not large. The advancing age, high total score on peak CT, peak eosinophils and ESR were associated with fibrotic change depicted by CT in patients recovering from COVID-19. An extended follow up by CT imaging and pulmonary function testing is necessary to fully assess fibrotic change in patients recovering from COVID-19.

## Data Availability Statement

The raw data supporting the conclusions of this article will be made available by the authors, without undue reservation.

## Ethics Statement

The studies involving human participants were reviewed and approved by Institutional review board of Tongji hospital, Tongji Medical College, Huazhong University of Science and Technology. Written informed consent from the participants' legal guardian/next of kin was not required to participate in this study in accordance with the national legislation and the institutional requirements. Written informed consent was not obtained from the individual(s), nor the minor(s)' legal guardian/next of kin, for the publication of any potentially identifiable images or data included in this article.

## Author Contributions

ZY and CC: study design, information collection, statistical analysis, and manuscript editing. All authors have read and approved the manuscript.

## Conflict of Interest

The authors declare that the research was conducted in the absence of any commercial or financial relationships that could be construed as a potential conflict of interest.

## Abbreviations

COVID-19: coronavirus disease 2019
CT: computerized tomography
GGO: ground-glass opacity
ESR: erythrocyte sedimentation rate
ECMO: extracorporeal membrane oxygenation
CRRT: continuous renal replacement therapy.

## References

[1] Zhu N Zhang D Wang W Li X Yang B Song J. A novel coronavirus from patients with pneumonia in China, 2019. N Engl J Med. (2020) 382:727–33. 10.1056/NEJMoa2001017 31978945 PMC7092803

[2] Dong D Tang Z Wang S Hui H Gong L Lu Y. The role of imaging in the detection and management of COVID-19: a review. IEEE Rev Biomed Eng. (2020). 10.1109/RBME.2020.2990959. [Epub ahead of print]. 32356760

[3] Simpson S Kay FU Abbara S Bhalla S Chung JH Chung M Radiological society of North America expert consensus statement on reporting chest CT findings related to COVID-19. Endorsed by the Society of Thoracic Radiology, the American College of Radiology, and RSNA. J Thorac Imaging. (2020) 2:e200152 10.1148/ryct.2020200152 PMC7255403 32324653

[4] Yang X Yu Y Xu J Shu H Xia J Liu H. Clinical course and outcomes of critically ill patients with SARS-CoV-2 pneumonia in Wuhan, China: a single-centered, retrospective, observational study. Lancet Respir Med. (2020) 8:475–81. 10.1016/S2213-2600(20)30079-5 32105632 PMC7102538

[5] World Health Organization Available online at: https://covid19.who.int/ (accessed May 14, 2020).

[6] Guan WJ Ni ZY Hu Y Liang WH Ou CQ He JX Clinical characteristics of coronavirus disease 2019 in China. N Engl J Med. (2020) 382:1708–20. 10.1056/NEJMoa2002032 32109013 PMC7092819

[7] Li K Wu J Wu F Guo D Chen L Fang Z. The clinical and chest CT features associated with severe and critical COVID-19 pneumonia. Invest Radiol. (2020) 55:327–31. 10.1097/RLI.0000000000000672 32118615 PMC7147273

[8] Xu X Yu C Qu J Zhang L Jiang S Huang D. Imaging and clinical features of patients with 2019 novel coronavirus SARS-CoV-2. Eur J Nucl Med Mol Imaging. (2020) 47:1275–80. 10.1007/s00259-020-04735-9 32107577 PMC7080117

[9] Huang C Wang Y Li X Ren L Zhao J Hu Y. Clinical features of patients infected with 2019 novel coronavirus in Wuhan, China. Lancet. (2020) 395:497–506. 10.1016/S0140-6736(20)30183-5 31986264 PMC7159299

[10] Chen N Zhou M Dong X Qu J Gong F Han Y. Epidemiological and clinical characteristics of 99 cases of 2019 novel coronavirus pneumonia in Wuhan, China: a descriptive study. Lancet. (2020) 395:507–13. 10.1016/S0140-6736(20)30211-7 32007143 PMC7135076

[11] Wang D Hu B Hu C Zhu F Liu X Zhang J. Clinical characteristics of 138 hospitalized patients with 2019 novel coronavirus-infected pneumonia in Wuhan, China. JAMA. (2020) 323:1061–9. 10.1001/jama.2020.1585 32031570 PMC7042881

[12] Liu J Chen T Yang H Cai Y Yu Q Chen J. Clinical and radiological changes of hospitalised patients with COVID-19 pneumonia from disease onset to acute exacerbation: a multicentre paired cohort study. Eur Radiol. (2020) 30:5702–8. 10.1007/s00330-020-06916-4 32385648 PMC7205907

[13] Pan F Ye T Sun P Gui S Liang B Li L. Time course of lung changes on chest CT during recovery from 2019 novel coronavirus (COVID-19) pneumonia. Radiology. (2020) 295:715–21. 10.1148/radiol.2020200370 32053470 PMC7233367

[14] Dai H Zhang X Xia J Zhang T Shang Y Huang R. High-resolution chest CT features and clinical characteristics of patients infected with COVID-19 in Jiangsu, China. Int J Infect Dis. (2020) 95:106–12. 10.1016/j.ijid.2020.04.003 32272262 PMC7136866

[15] Zhou S Wang Y Zhu T Xia L. CT features of coronavirus disease 2019 (COVID-19) pneumonia in 62 patients in Wuhan, China. AJR Am J Roentgenol. (2020) 214:1287–94. 10.2214/AJR.20.22975 32134681

[16] Antonio GE Wong KT Hui DS Wu A Lee N Yuen EH. Thin-section CT in patients with severe acute respiratory syndrome following hospital discharge: preliminary experience. Radiology. (2003) 228:810–5. 10.1148/radiol.2283030726 12805557

[17] Das KM Lee EY Singh R Enani MA Al Dossari K Van Gorkom K. Follow-up chest radiographic findings in patients with MERS-CoV after recovery. Indian J Radiol Imaging. (2017) 27:342–9. 10.4103/ijri.IJRI_469_16 29089687 PMC5644332

[18] Sun P Qie S Liu Z Ren J Li K Xi J Clinical characteristics of hospitalized patients with SARS-CoV-2 infection: a single arm meta-analysis. J Med Virol. (2020) 92:612–7. 10.1002/jmv.25735 32108351 PMC7228255

[19] World Health Organization Clinical Management of COVID-19 (2020). Available online at: https://www.who.int/publications-detail-redirect/clinical-management-of-covid-19 (accessed November 9, 2020)

[20] General Office of National Health Committee Notice on the Issuance of a Program for the Diagnosis and Treatment of Novel Coronavirus (2019-nCoV) Infected Pneumonia (Trial Seventh Edition) (2020-03-03). Available online at: http://www.nhc.gov.cn/yzygj/s7653p/202003/46c9294a7dfe4cef80dc7f5912eb1989.shtml (accessed March 3, 2020).

[21] Yu N Li W Kang Q Xiong Z Wang S Lin X Clinical features and obstetric and neonatal outcomes of pregnant patients with COVID-19 in Wuhan, China: a retrospective, single-centre, descriptive study. Lancet Infect Dis. (2020) 20:559–64. 10.1016/S1473-3099(20)30176-6 32220284 PMC7158904

[22] Hsu HH Tzao C Wu CP Chang WC Tsai CL Tung HJ. Correlation of high-resolution CT, symptoms, and pulmonary function in patients during recovery from severe acute respiratory syndrome. Chest. (2004) 126:149–58. 10.1378/chest.126.1.149 15249456 PMC7094423

[23] Ooi GC Khong PL Muller NL Yiu WC Zhou LJ Ho JC. Severe acute respiratory syndrome: temporal lung changes at thin-section CT in 30 patients. Radiology. (2004) 230:836–44. 10.1148/radiol.2303030853 14990845

[24] Zhang L Zhang C Dong F Song Q Chi F Liu L. Combined pulmonary fibrosis and emphysema: a retrospective analysis of clinical characteristics, treatment and prognosis. BMC Pulm Med. (2016) 16:137. 10.1186/s12890-016-0300-7 27809901 PMC5093954

[25] Kolahian S Fernandez IE Eickelberg O Hartl D. Immune mechanisms in pulmonary fibrosis. Am J Respir Cell Mol Biol. (2016) 55:309–22. 10.1165/rcmb.2016-0121TR 27149613

[26] Horowitz JC Thannickal VJ. Mechanisms for the Resolution of Organ Fibrosis. Physiology (Bethesda). (2019) 34:43–55. 10.1152/physiol.00033.2018 30540232 PMC6383633

[27] Sartorius A Lu Q Vieira S Tonnellier M Lenaour G Goldstein I. Mechanical ventilation and lung infection in the genesis of air-space enlargement. Crit Care. (2007) 11:R14. 10.1186/cc5680 17274806 PMC2147711

[28] Milliron B Henry TS Veeraraghavan S Little BP. Bronchiectasis: mechanisms and imaging clues of associated common and uncommon diseases. RadioGraphics. (2015) 35:1011–30. 10.1148/rg.2015140214 26024063

[29] Wynn TA. Integrating mechanisms of pulmonary fibrosis. J Exp Med. (2011) 208:1339–50. 10.1084/jem.20110551 21727191 PMC3136685

[30] Fulkerson PC Fischetti CA Rothenberg ME. Eosinophils and CCR3 regulate interleukin-13 transgene-induced pulmonary remodeling. Am J Pathol. (2006) 169:2117–26. 10.2353/ajpath.2006.060617 17148674 PMC1762480

[31] Cabrera-Benitez NE Parotto M Post M Han B Spieth PM Cheng WE. Mechanical stress induces lung fibrosis by epithelial-mesenchymal transition. Crit Care Med. (2012) 40:510–7. 10.1097/CCM.0b013e31822f09d7 21926573 PMC5061566

